# BioCreative V track 4: a shared task for the extraction of causal network information using the Biological Expression Language

**DOI:** 10.1093/database/baw067

**Published:** 2016-07-09

**Authors:** Fabio Rinaldi, Tilia Renate Ellendorff, Sumit Madan, Simon Clematide, Adrian van der Lek, Theo Mevissen, Juliane Fluck

**Affiliations:** 1 Institute of Computational Linguistics, University of Zurich, Zurich, Switzerland; 2 Fraunhofer Institute for Algorithms and Scientiﬁc Computing, Schloss Birlinghoven, Sankt Augustin, Germany

## Abstract

Automatic extraction of biological network information is one of the most desired and most complex tasks in biological and medical text mining. Track 4 at BioCreative V attempts to approach this complexity using fragments of large-scale manually curated biological networks, represented in Biological Expression Language (BEL), as training and test data. BEL is an advanced knowledge representation format which has been designed to be both human readable and machine processable. The specific goal of track 4 was to evaluate text mining systems capable of automatically constructing BEL statements from given evidence text, and of retrieving evidence text for given BEL statements. Given the complexity of the task, we designed an evaluation methodology which gives credit to partially correct statements. We identified various levels of information expressed by BEL statements, such as entities, functions, relations, and introduced an evaluation framework which rewards systems capable of delivering useful BEL fragments at each of these levels. The aim of this evaluation method is to help identify the characteristics of the systems which, if combined, would be most useful for achieving the overall goal of automatically constructing causal biological networks from text.

## Introduction

Biological networks with a structured syntax are a powerful way of representing biological information and knowledge. Well-known examples of standards to formally represent biological networks are the Systems Biology Markup Language (SBML) (1), the Biological pathway exchange language (BioPAX) (2) and the Biological Expression Language (http://www.openbel.org/) (BEL) (3). These approaches are not only designed for the representation of biological events, but they are also intended to support downstream computational applications. In particular, BEL is gaining ground as the de-facto standard for systems biology applications because it combines the power of a formalized representation language with a relatively simple syntax that allows an easy interpretation of BEL statements by a trained domain expert.

As part of an on-going systems biology method verification, the sbvIMPROVER initiative is a platform providing datasets and assessments of various methodologies in systems biology (4,5). One of the more recent challenges was a large-scale crowdsourcing approach for the verification of biological networks (6–9), called Network Verification Challenge (NVC) (10). The NVC supports community-based verification and extension of biological relationships based on peer-reviewed literature evidence. At present, 50 biological networks have been curated, all available in BEL format, with supporting evidence text in form of a sentence or section and a PubMed identifier.

Using data provided by the NVC, we designed a novel text mining challenge aimed at evaluating the capability of text mining system to retrieve useful fragments of biological networks. This novel challenge was organized as ‘track 4’ within the context of the 5th edition of the well-known BioCreative series of shared tasks. BioCreative is a community-organized framework which provides a rigorous evaluation framework for biomedical text mining technologies. We provided training and test corpora selected from the biological networks manually curated in the NVC, thus assuring high quality of the data (11). The complexity of the problem led us to design an evaluation framework capable of giving partial credit to systems able to retrieve useful fragments of BEL statements, even in cases where the complete BEL statement could not be identified correctly. The reasoning behind this approach is that such fragments could be useful in a semi-automated environment to help guide manual curators of BEL statements.

The goal of the challenge that we proposed was to assess the utility of such tools either for the automated annotation and network expansion, or their suitability as supporting tools for assisted curation. The challenge was organized into two tasks, evaluating two complementary aspects of the problem:

***Task 1: Given an evidence text, generate the corresponding BEL statements***.

***Task 2: Given a BEL statement, provide at most 10 additional evidence texts***.

In the rest of this paper we first provide an overview of related work (‘Related work’ section). We follow with a description of the training and test material used in the challenge, and of the evaluation framework (‘Materials and methods’ section), then illustrate in detail the official results achieved by the participating systems (‘Results’ section), and conclude with a description of the best participating systems (‘Participating systems’ section).

## Related work

### Biomedical shared tasks

The field of biomedical text mining has a long-standing tradition of systematic and rigorous evaluation through community-organized shared tasks. Probably the best well-known of such evaluations is the BioCreative conference series (12). Similar well-known competitive evaluations that have had a major impact on the field include the BioNLP series (13), i2b2 (14), CALBC (15), CLEF-ER (16), DDI (17) or BioASQ (18).

Each of these competitions targets different aspects of the problem, sometimes with several subtasks, such as detection of mentions of specific entities (e.g. genes and chemicals), detection of protein interactions, assignment of Gene Ontology tags (BioCreative), detection of structured events (BioNLP), information extraction from clinical text (i2b2), large-scale entity detection (CALBC), multilingual entity detection (CLEF-ER), drug-drug interactions (DDI), question answering in biology (BioASQ).

First organized in 2004, BioCreative provides the most reliable platform for the evaluation and comparison of biomedical text mining systems. Each BioCreative conference provides the opportunity to discuss the results of a small set of challenges that are run in the previous months. Several biomedical problems of extraction of information from the biomedical literature have been examined within the scope of the five editions of the challenge, such as for example: recognition of gene mention (19), normalization of gene mention to standardized database identifiers (20), assignment of GO terms (21), detection of protein-protein interactions (22).

The organizers of each of these challenges typically provide several months in advance a dataset which has been manually verified for accuracy. The participants are given a section of that data as ‘training corpus’, while another section is held by the organizers and used to measure accurately the capability of the participating systems to reproduce the annotations provided in the training data. Such rigorous evaluation provides a reliable platform for the comparison of competing techniques, and enables scientific progress through exchange of best practices.

### Biological expression language

**Figure 1:**
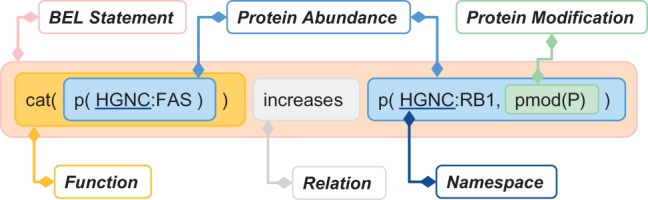
Example of BEL statement (The ‘cat’ function representing catalytic activity was considered in our evaluation as equivalent to ‘act’ (activity), see Table 3 for details.).

The biological expression language (BEL) is designed to represent scientific findings in the field of life sciences in a form that is not only computable but also easily editable by humans. The findings are captured through causal and correlative relationships between entities in the format of BEL statements. One example of a BEL statement is presented in Figure 1.

**Figure 2:**
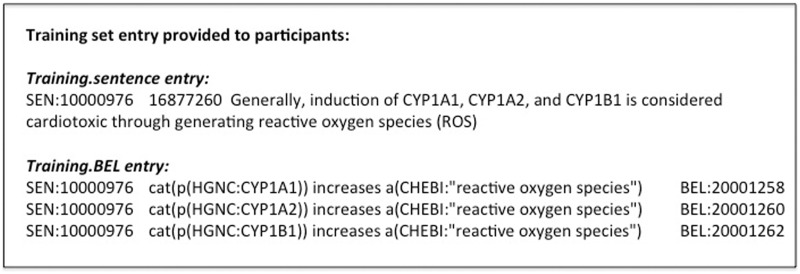
Training data example.

Publication references are provided as supporting information for each statement. Most BEL statements represent relationships between one BEL term and another BEL term or a subordinate BEL statement. Example BEL statements related to an evidence sentence are shown in Figure 2. The statements typically encode a semantic triple (subject, predicate and object). The predicate is one of the BEL relationship types describing the relationship between the subject and object. For track 4, we selected in particular causal relationships as shown in Table 1.

**Table 1.** BEL Relationships evaluated in Track 4

Relationship—long form	Short form	Example
**Decreases**	−|	a(CHEBI:‘brefeldin A’) -| p(HGNC:SCOC)
**directlyDecreases**^1^	=|	p(HGNC:TIMP1) =| act(p(HGNC:MMP9))
**Increases**	−>	p(MGI:Bmp4) -> p(MGI:Acta2)
**directlyIncreases**^2^	= >	p(HGNC:VEGFA) = > act(p(HGNC:KDR))

1 In the challenge, decreases was accepted in place of directlyDecreases.

2 In the challenge, increases was accepted in place of directlyIncreases.

The specification of BEL allows for an easy integration of external vocabularies and ontologies. BEL adopts a concept of namespaces (e.g. CHEBI) to normalize entities in a flexible way. By applying namespace prefixes a user can establish references to elements of the specific vocabulary (e.g. CHEBI:‘nitric oxide’). Currently, BEL offers >20 different namespaces. For simplification purposes the dataset used in track 4 was restricted to a selection of 6 namespaces (c.f. Table 2). Different namespaces have different abundance and process functions associated with them These ‘functions’ in BEL terminology serve to assign a type to the object that they apply to (gene, protein, biological process, pathological process, etc.). They should not be confused with functions used to modify entities (e.g. degradation, translocation). BEL terms are formed using these BEL functions together with the namespaces and the associated identifiers, e.g. *a(CHEBI:‘nitric oxide’)*. An overview of short and long function names associated to namespaces can be found in Table 2. In order to find equivalences between the entities of different namespaces, a range of equivalence resources are provided at the OpenBEL website (https://github.com/OpenBEL/openbel-framework-resources/tree/latest/equivalence).

**Table 2.** Overview of Track 4 namespaces and associated functions

Namespace Identifier	Description	Associated Entities	BEL Functions	Function Longform	BEL Term Example
**HGNC** (HUGO Gene Nomenclature Committee)	Standard approved gene symbols and synonyms for Humans, used to specify genes, microRNA, RNA and proteins	Human Genes, microRNA, RNA, proteins	p(),g(),r(),m(),	proteinAbundance**()**geneAbundance(),rnaAbundance(),microRNAAbundance(),	p(HGNC:MAPK14)
**MGI** (Mouse Genome Informatics)	Standard approved gene symbols and synonyms for Mouse, used to specify genes, microRNA, RNA and proteins	Mouse Genes, microRNA, RNA, proteins	p(),g(),r(),m(),	Same as above	p(MGI:Mapk14)
**EGID** (Entrez Gene Identifiers)	Genes, microRNA, RNA and proteins of Homo sapiens, Mus musculus and Rattus norvegicus.	Genes, microRNA, RNA, proteins	p(),g(),r(),	Same as above	p(EGID:1432)
**GOBP** (Gene Ontology Biological Process)	Gene Ontology database for biological processes referenced through the standard name.	Biological Processes	bp()	**biologicalProcess()**	bp(GOBP:‘cell proliferation’)
**MESHD** (Medical Subject Headings Diseases)	U.S. National Library of Medicine provided vocabulary for disease. Namespace provides the Main Heading for each disease in the Diseases [C] tree. These identifiers can be used to specify pathologies.	Diseases, Pathologies	path()	**pathology()**	path(MESHD:Hyperoxia)
**CHEBI** (Chemical Entities of Biological Interest (ChEBI) database)	Chemical Entities referenced through the standard name for each compound.	Chemicals	a()	**abundance()**	a(CHEBI: lipopolysaccharide)

Information about the state (e.g. transformation, translocation or molecular activity) in which entities are found, is encoded as functions, which take BEL terms as arguments (e.g. ‘cat’ in Figure 1). An overview of selected functions for the task is provided in Table 3.

**Table 3.** Overview of selected functions

Function	Function Type	Example
complex() *complexAbundance()*	Abundances	(complex(p(MGI:Itga8),p(MGI:Itgb1))) -> bp(GOBP:‘cell adhesion')
pmod() *proteinModification()*	Modifications	p(MGI:Cav1,pmod(P)) -> a(CHEBI:‘nitric oxide')
deg() *degradation()*	Transformations	p(MGI:Lyve1) -> deg(a(CHEBI:‘hyaluronic acid'))
tloc() *translocation()*	Transformations	a(CHEBI:‘brefeldin A') -> tloc(p(MGI:Stk16))
act() *molecularActivity()*	Activities	complex(p(MGI:Cckbr),p(MGI:Gast)) -> act(p(MGI:Prkd1))

## Materials and methods

### Training and test data

The BioCreative track 4 dataset (including training, sample and test set) was selected from two corpora provided by Selventa and the sbv IMPROVER Network Verification Challenge (https://bionet.sbvimprover.com/). These resources contain BEL statements along with associated citations and evidence text snippets. The selection and re-annotation processes used to create the final dataset are described in detail in (11). In short, the BEL_Extraction training corpus is restricted in an automated way to the entity classes, functions and relationships selected for the BioCreative BEL track. In addition, the associated evidence text snippets are limited in length to contain at most two sentences. For the creation of the BEL_Extraction training corpus, evidence texts were randomly selected and all associated BEL statements were extracted. This corpus served as a training set for both tasks: the extraction of BEL statements from the evidence texts (task 1) and the retrieval of evidence sentences for the given BEL statements (task 2). Overall, it contains 6353 unique evidence texts and 11 066 BEL statements. The dominant category types in the training set are the following: 87% of the terms are proteins, 69% of the functions are activations and 73% of the relations express an increase.

In addition, a smaller corpus, the BEL_Extraction sample corpus was provided for proper evaluation during development. This dataset was manually re-annotated to restrict it to BEL statement–evidence pairs where the evidence contains sufficient information to allow the extraction of the full statement. It is composed of 191 sentences with 296 BEL statements.

Finally, the BEL_Extraction test corpus is used for the evaluation of automated predictions. For this dataset, we verified that the data were not publicly available. It was re-annotated in a similar way as the sample set. Based on results of first prediction evaluations, we added a number of missing statements to the test set before it was used within the final BioCreative evaluation process. The test set comprises 105 sentences accompanied by 202 statements. The class distribution for both smaller datasets (sample set and test set) are similar to the training set except for the function level where activation covers only 46% of all cases.

For task 2, the test data were composed of 100 BEL statements. Only BEL statements which satisfied the following conditions were selected, (i) the BEL statement-evidence pair was not included in the BEL extraction corpora described above, and (ii) the accompanied evidence text could be found in Medline. In this way, we verified the presence of at least one positive Medline abstract comprising an evidence text for every statement.

### Supporting resources

The participants were provided with a range of supporting resources and a comprehensive documentation (http://wiki.openbel.org/display/BIOC/Biocreative+Home), containing a description of the format and detailed explanation of the evaluation process. The evaluation method on the different levels of a single BEL statement, as described in ‘the Results section’, was illustrated using a set of concrete example submissions as reference. Additionally, an evaluation interface (http://bio-eval.scai.fraunhofer.de/cgi-bin/General_server.rc) was provided for the participants to test their generated statements during the development phase. The interface is described in detail in ‘the Evaluation method’ section.

**Figure 3:**
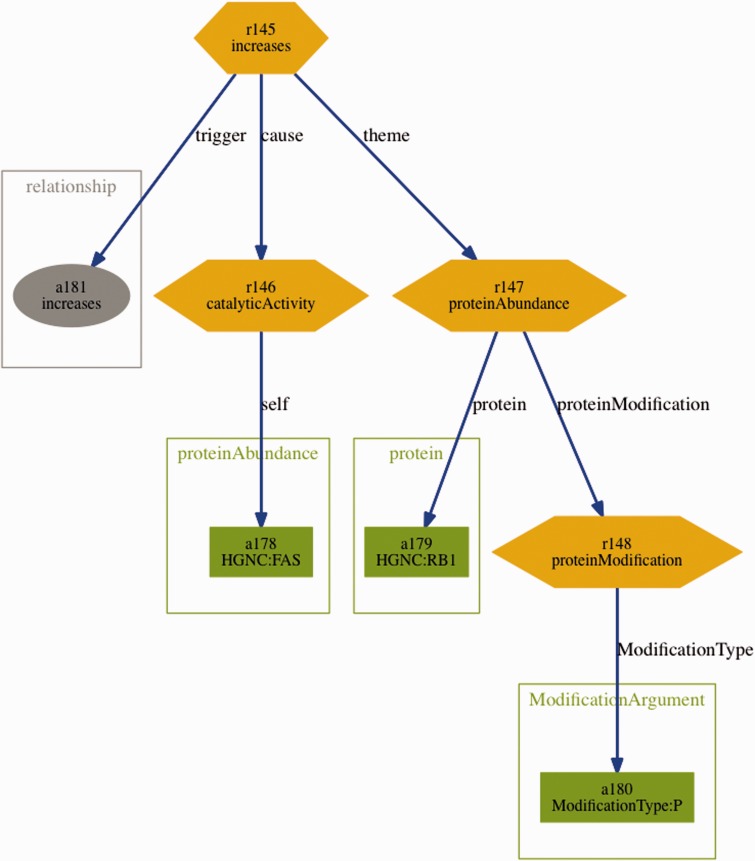
Visualization of the BEL statement ‘*cat(p(HGNC:FAS)) increases p(HGNC:RB1,pmod(P))*’ derived from the sentence ‘Fas stimulation of Jurkat cells is known to induce p38 kinase and we find a pronounced increase in Rb phosphorylation within 30 min of Fas stimulation’.

Further supporting resources included the BEL statements from the training and sample set in BioC format. These were generated automatically using a converter based on the official ruby-based BEL parser (http://www.openbel.org/tags/bel-parser-belrb) and an open-source BioC ruby module (https://github.com/dongseop/simple_bioc) (23). Furthermore, a tab-separated format containing all fragments of the BEL statements (terms, functions and relations) was generated from the sample and training set, using the same BEL parser mentioned above. Finally, graph visualizations representing the structure of the BEL statements were automatically derived from the BioC format. An example for such visualization can be seen in Figure 3.

### Evaluation method

The automated extraction of relationships from text, and the generation of their BEL representation, is a complex task due to the different entity, function and relationship types. Furthermore, not all information encoded in the expert-generated BEL statements can be directly found in the evidence text provided as training data, since curators might use some degree of interpretation. Besides, a certain level of arbitrariness is involved in the decision of what information from a sentence has to be encoded in the corresponding BEL statement. Additionally, there can be several different ways to correctly encode the selected information in BEL.

Therefore, our aim was to design an evaluation scheme that is liberal enough to give partial credit if a submitted BEL statement is partially correct, compared to the gold standard and fine-grained enough to allow for an exact and detailed evaluation. We reached this aim by designing the evaluation scheme in a way to allow for simplification of BEL statements and by providing a cascade model for evaluation, which considers different structural levels of BEL statements. On all of these levels, evaluation scores were calculated by using precision, recall and *F*-measure as evaluation metrics. Since BEL is a formal language, BEL statements or fragments provided by the participants must be syntactically correct to be accepted for evaluation.

#### Evaluation simplifications

A range of simplifications was introduced in the evaluation process in order to grant the evaluation scheme a higher degree of fairness and flexibility. An additional advantage is that as we merge items that are similar but considered distinct in BEL, we automatically provide more training material for each of them.

The first simplification consists in entity mapping. The dataset includes three different namespaces (EGID, MGI, HGNC), associated to the protein abundance function p(). In order to be able to choose the correct namespace for a specific protein, a system would need to include a step of organism disambiguation. However, we did not expect the participants to perform organism disambiguation, given the limited context provided as evidence text, instead we accepted all three namespaces, and mapped them to the HGCN namespace, accepting all equivalent cases.

Second, function evaluation is simplified by mapping activity functions, such as *kin()*, *tscript()* and *cat()*, to the more generic *act()* function. In this way we did not expect the participating systems to discover subtle distinctions between different types of molecular activity. A system is given credit if it is able to discover any kind of molecular activity. Furthermore, the modification function *pmod()* and the translation function *tloc()* are reduced in their number of arguments. *pmod(P)* is evaluated without the position and amino acid information and the *tloc()* function is evaluated without information of the location.

Third, the evaluation scheme does not differentiate between unspecific and direct relationship types. This means that *increases* and *directlyIncreases* are treated as equal. The same is true for *decreases* and *directly Decreases*.

Finally, placeholders can be used for terms and relationships. Placeholder terms can be used as formally correct dummy entities (*p(‘PH:placeholder’)*) to provide arguments to BEL functions and relationships. The relationship type ‘*association*’ (short form ‘–’) is provided as a placeholder for all cases where the relationship type and/or the direction is unknown. Placeholders count as false negatives but not as false positives, and therefore, only influence recall but not precision. Therefore, placeholder allow participants to formulate syntactically complete BEL statements even if their system cannot find all the information that would be necessary to build them.

#### The validation and evaluation web service

During the development phase, the participants were invited to evaluate their predictions through an evaluation interface (http://bio-eval.scai.fraunhofer.de/cgi-bin/General_server.rc). This interface was developed with the programming language Perl and runs as a CGI script under a web server. The interface provides two main functionalities – BEL statement validation and task 1 evaluation. The BEL statement validator validates the input BEL statements submitted by a user with respect to their formal correctness, as described above. The system uses the Java-based *OpenBEL Framework (version 2.0.1)* to validate the BEL statements. If statements are invalid, users are given the chance to find and correct the errors. For this purpose, errors are visualized by the web interface.

**Figure 4:**
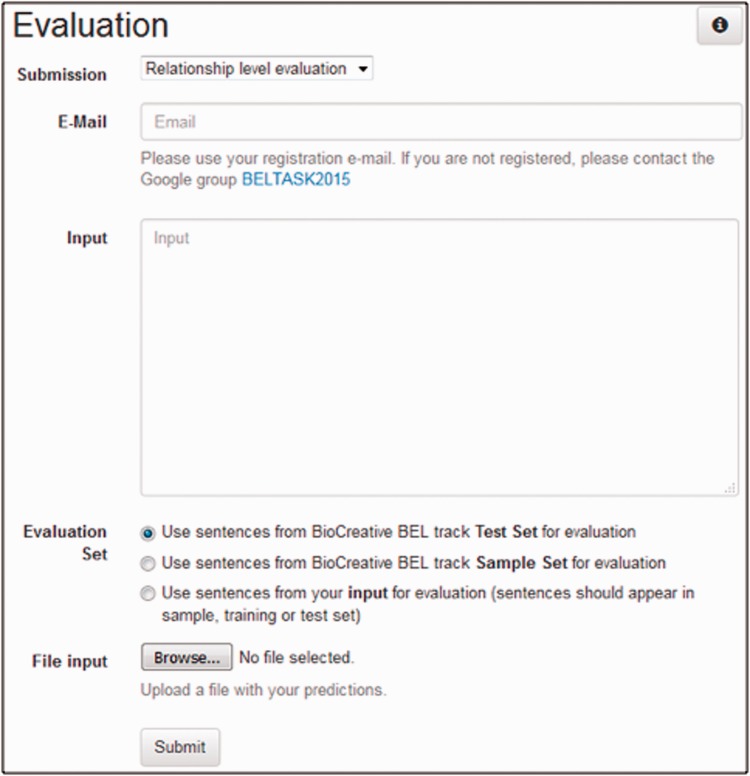
A screenshot of the evaluation user interface of task 1.

The users can evaluate the predictions of their system using the task 1 evaluation web interface. Figure 4 shows a screenshot of the user interface. To start the evaluation, a user has to provide the input BEL statements to be evaluated as well as the submission type and an e-mail address. The submission type decides on which structural level (term, function and relationship as described below) the input will be evaluated. A user can choose between two different ways for providing input. Either a file with predictions can be uploaded to the service or predictions can be submitted directly by using the text field.

For the choice of the evaluation set, we provide three different options: *sample set*, *test set* and *evaluation set of your choice*. The sample and test options use the task sample and test set respectively. Through the third option *evaluation set of your choice* a user can define a custom evaluation set. The gold standard for the sentences occurring in the user input will be used for evaluation. The only restriction is that the sentences should appear in the dataset (training, sample or test set) of task 1. This option can be useful in an n-fold cross-validation setting.

**Figure 5:**
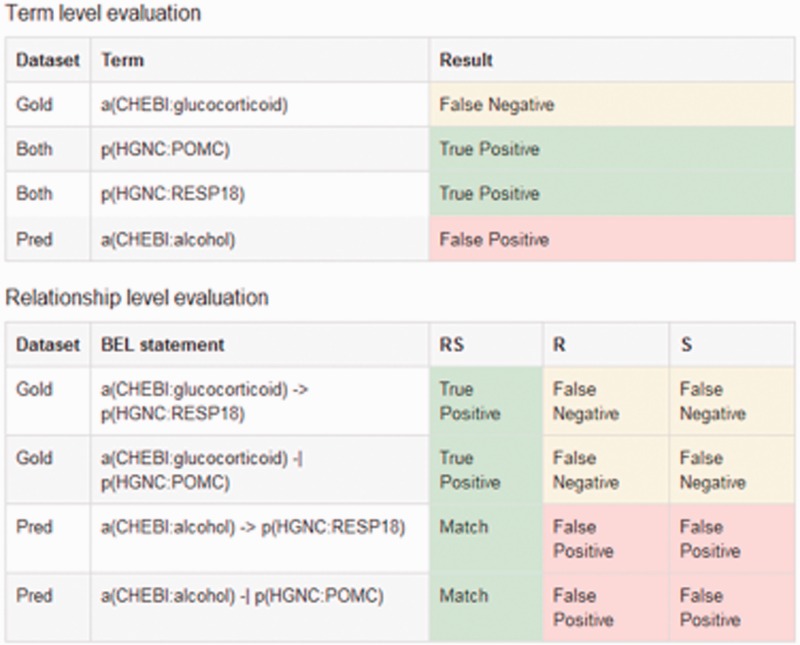
An example output of the sentence-based evaluation. The screenshot contains the detected true positive (green), false positive (red) and false negatives (yellow) entries for the term and relationship level.

The output of the evaluation page shows results per evidence text and an overall performance statistics. The overall performance statistics contains values for true positives, false positives, false negatives and the evaluation metrics recall, precision and F-score for all different structural levels. The statistics includes the performance statistics for each evidence text. In addition, further information is provided, such as the evidence text itself, the gold standard BEL statement derived from the chosen evaluation set and the predicted BEL statements taken from the user's input. Furthermore, true positive, false positive and false negative entries for the various structural levels are displayed, as can be seen in Figure 5. The overall performance statistics shows the combination of the results of all evidence texts.

### Evaluation of task 1 on different structural levels

**Figure 6:**
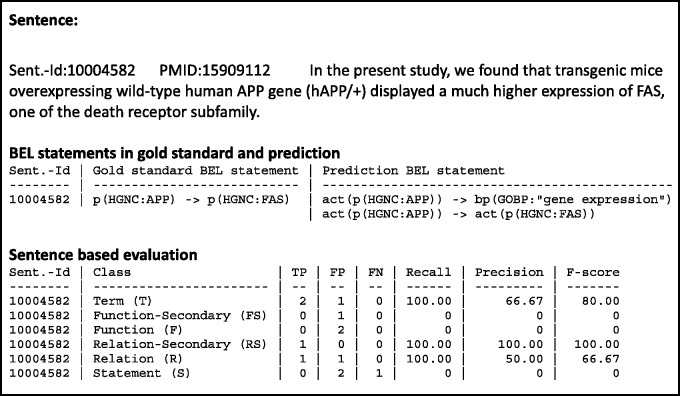
An example result page of a candidate evaluation. The example shows the candidate sentence, with the gold standard and the predicted BEL statements. The evaluation scores are shown for all primary and secondary levels.

In the cascade evaluation model, different levels of performance are evaluated associated to the different structural levels of BEL statements, namely the BEL terms, BEL functions, BEL relationships and, ultimately, the full BEL statements. This evaluation scheme is based on the intuition that participating systems might differ in their individual strengths and weaknesses and might show a strong performance on one or several of these levels. Furthermore, discovering BEL statements that are fully correct in all their components is a very hard task. For this reason, we designed the evaluation scheme to enable us to give credit to partially correct BEL statement as well. A submitted syntactically valid full BEL statement is automatically cut into its fragments to enable this kind of evaluation. Moreover, submissions could be made on different levels. A maximum number of three submissions were allowed in task 1. An overview of all evaluation levels can be seen in Table 4. An example of a candidate evaluation is shown in Figure 6.

**Table 4.** Overview of the different evaluation levels with examples

BEL Statement	p(HGNC:BCL2A1) decreases bp(GOBP:’apoptotic process')	act(p(MGI:Hras)) increases p(MGI:Mmp9)
**Evidence Sentence**	*We demonstrate that the Bfl-1 protein suppresses apoptosis induced by the p53 tumor suppressor protein in a manner similar to other Bcl-2 family members such as Bcl-2, Bcl-xL and EBV-BHRF1.*	*Cells with activated ras demonstrated high level of expression of 72-kDa metalloproteinase (MMP-2, gelatinase A) and 92-kDa metalloproteinase (MMP-9, gelatinase B) compared with cells containing SV40 large T antigen alone.*

**Term-level Evaluation (T)**	p(HGNC:BCL2A1)	p(MGI:Hras)
	bp(GOBP:’apoptotic process')	p(MGI:Mmp9)

**Function-level Evaluation (F)**	–	act(p(MGI:Hras))
**Secondary Function-level Evaluation (Fs)**		*For secondary: only the function itself is evaluated regardless of the argument*

**Relationship-level Evaluation (R)**	p(HGNC:BCL2A1) decreases bp(GOBP:’apoptotic process')	p(MGI:Hras) increases p(MGI:Mmp9)
**Secondary Relationship-level Evaluation (Rs)**		*For secondary: two of the three elements of the relation (arguments and relation type) have to be correct*

**Full-statement evaluation (S)**	p(HGNC:BCL2A1) decreases bp(GOBP:’apoptotic process')	act(p(MGI:Hras)) increases p(MGI:Mmp9)

#### Evaluation on the term level

On the term level, the correctness of all BEL terms that are part of BEL statements is evaluated. This includes the entities, namespaces and associated abundance or process functions. All these parts of a BEL term need to be correct to credit a true positive. Partially correct BEL terms are considered as false positive. However, as mentioned above, organism disambiguation was not expected. Furthermore, on the term level, placeholder entities were introduced to allow the submission of incomplete information. This ensures that even if entity or namespace information is missing, a BEL term is still formally correct. Instead of exact namespaces and identifiers, placeholders were accepted in the format ‘PH:placeholder’. As discussed previously, placeholders allow participants to submit syntactically correct statements in the absence of a correct entity, without being double penalized in precision and recall, as placeholders influence only recall (one false negative) but not precision (no false positive).

#### Evaluation on the function level

On the function level, the correctness of the functions within BEL statements is evaluated. Functions were only accepted for evaluation if they included their argument BEL terms. In order to allow for a more fine-grained evaluation of function-argument units, function evaluation was divided in two sub-levels: on the primary sub-level, correct arguments are expected and no credit was given if incorrect arguments were provided. The special function *complex()* was considered as valid if at least one of its arguments was correct. On the secondary level, only the correctness of a function on its own was evaluated, regardless of the correctness of its arguments. This means that on the secondary level, functions could achieve a full score even if they contain placeholders as arguments or any other incorrect BEL terms.

Simplifications on the function level were made by mapping all activity functions into act(), as previously described in ‘Evaluation simplifications’ section, and by restrictions concerning additional arguments other than BEL terms.

#### Evaluation on the relationship level

On the relationship level, the core relation within each BEL statement is evaluated. All components of a BEL relationship are taken into account. The correctness of the BEL terms (subject and object) as well as the type of relationship is considered. Functions are not evaluated at this level, and are therefore discarded if included in the submitted statements.

Evaluation on the relationship level is further divided into two sub-levels: the primary level requires all three components of a relationship to be correct, that is the BEL terms as argument of a relation, as well as the relationship type. If one of these components is incorrect, no credit is given. In the special case of the *complex()* function, one correct function argument being in a correct relationship is sufficient for a positive score. On the secondary level, credit is given in all cases where two components are correct. This means either a correct relationship type is found together with at least one correct argument, or both subject and object are correct even when the relationship type is incorrect, or the relationship type *‘association’* (short form ‘–’) was used in place of the correct relationship. This placeholder could be used in all cases where the relationship type and/or direction could not be determined.

#### Evaluation on the full statement level

On the full statement level, a submission is credited a full score if it is equal to the BEL statement in the gold standard, with simplifications applied. The submission of incomplete BEL statements, even though it could achieve a higher score on other levels, had the effect of lowering scores on the full statement level.

If a full statement was correct but BEL terms or functions are expressed as placeholders instead of namespaces and identifiers, only a FN (false negative) but no FP (false positive) was counted. This was done in order to give credit to systems capable of retrieving partially correct information: the placeholder enables them to increase their recall, without penalizing their precision.

### Evaluation of task 2

For the retrieval of evidence for the given BEL statements, we accepted evidence texts from Medline abstracts as well as from the PMC full text corpus. As a single piece of evidence text, a maximum of two sentences could be proposed. Submissions with longer text size were discarded. This size restriction was established to limit the curator workload because all submissions for task 2 had to be evaluated manually. Up to 10 different pieces of evidence were evaluated for each BEL statement. The evaluators had to decide whether the provided evidence text could be considered as a source for a given BEL statement. Three different criteria were applied in evaluating the sentences: full, relaxed and context. For the *full criterion* every single information of the BEL statement has to be represented in the evidence. For the *relaxed criterion*, the evidence is counted as true positive when more context information is necessary to decide if the evidence contains all the BEL information. In the example evidence *‘The M-CSF-induced macrophages resulted in enhanced foam cell formation, which could be inhibited by monoclonal antibodies to CD36’* it is not perfectly obvious that M-CSF increases CD36 but it cannot be ruled out. Such an evidence sentence would not be annotated as full true but relaxed true evidence. The *context annotation* criterion is rather weak: to be considered as correct, the evidence must contain all entities and at least a relationship for one of the entities. In a post-Biocreative corpus annotation step, the guidelines for this annotation method were refined and the context criterion discarded. We refer to (11) ****for further details.

## Results

### Task 1: Given textual evidence for a BEL statement, generate the corresponding BEL statement

Five teams contributed information extraction systems for task 1. Each team was permitted to provide up to 3 runs, allowing them to test different configurations of their systems. Additionally, we performed the evaluation in two stages. In stage 1, participants had to detect named entities from the provided evidence. In stage 2, the ‘gold standard’ named entities were provided.

Table 5 shows the results for this task in stage 1, where the teams had to provide their own term recognition. The results are color-coded in shades of green according to the values of *F*-score (*F*), the main evaluation criterion and supplemented by the values for precision (*P*) and recall (*R*). The best results for each evaluation metrics are marked up in bold.

**Table 5.** Evaluation of stage 1 of task 1 (prediction of BEL statements without gold standard entities)

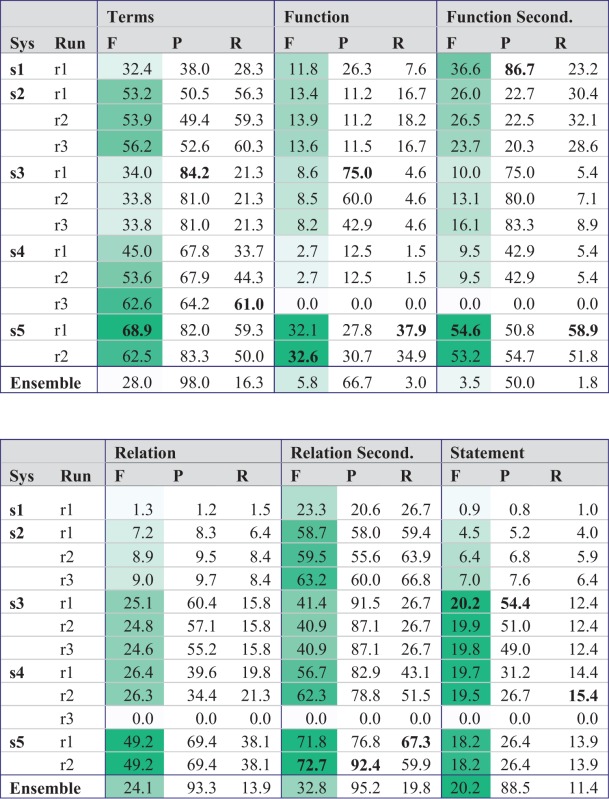

For the full statement level, the best system (s3) achieved 20% *F*-measure, which illustrates the difficulty of this highly structured prediction task. System s4 and s5 had a similar performance, although their results were quite different on other evaluation levels, e.g. the term level. Obviously, the performance on the function level does not correlate well with the performance of the full statement level. One of the reasons is the lack of functions in 39 statements out of 105 test set statements. Furthermore, high scores on the relation level do not necessarily correlate with high scores on the full statement level. On the secondary relation level where only two out of three elements of the relationship have to be correct, up to 72.7% *F*-score were achieved.

In a final step, we explored whether the performance can be enhanced through ensemble solutions. Considering all submitted statements of the five teams, the recall reaches 32.2% (best individual system run achieves 15.4%) but the precision drops to 9.2%. As result, the F-measure of 14.3% is substantially lower compared to the best individual system and therefore not a viable solution (This hypothetical ensemble system is not shown in the result tables.).

An ensemble system that considers all statements predicted by at least 2 different systems performs on F-measure level on par with the best individual system (c.f. Table 5). However, precision was gained at the expense of lower recall. Overall, the upper limit on recall for any ensemble system is quite low: for 62 sentences (59%), no participating system could find any correct BEL statement. On the level of relations, 42 sentences (40%) had no true positive. Further analysis is needed for understanding why all system failed consistently in a substantial number of the cases.

Table 6 shows the results for stage 2 of task 1 where the gold standard terms of the test set were made available to the teams. Most systems strongly benefit and improve on the level of full statements. These results prove again that high-quality relation extraction crucially depends on high-quality term recognition. With this setting, system s3 can compensate its rather low recall on the level of terms and can reach the best *F*-measure of 35.2% on the level of full statements. In this stage, an ensemble system considering all statements predicted by at least 2 different systems outperforms the best individual system by almost 4%. The number of sentences where no system predicts any correct BEL statement dropped from 62 to 44 sentences (42%). On the level of relations, 19 sentences still had no true positive.

**Table 6.** Evaluation of stage 2 of task 1 (prediction of BEL statements with gold standard entities)

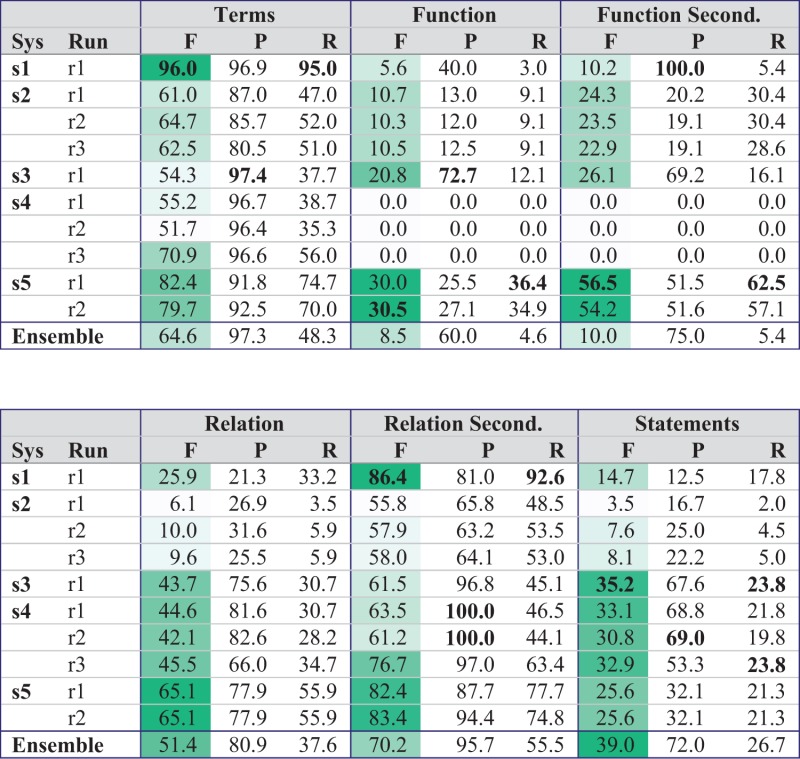

### Task 2: Given a BEL statement, provide at most 10 additional evidence sentences

Only one team participated in task 2. The correctness of the provided evidence sentences (up to 10 sentences for each BEL statement) was evaluated manually and rated on three different levels of strictness:

1. Full: Relationship is fully expressed in the sentence.
2. Relaxed: Relationship can be extracted from the sentence if context sentences or biological background knowledge are taken into account.
3. Context: The sentence provides a valid context for the relationship, the entities are described by the sentence but the correct relation may not be expressed.

The system provided 806 evidence sentences for 96 BEL statements (mean 8.3 sentences per statement with a standard deviation 3.0). For 72 BEL statements, there was at least one entirely correct evidence sentence, for 78 statements at least one sentence meeting the relaxed evaluation conditions, and for 81 a sentence meeting the contextual conditions. Table 7 shows the detailed numbers for true positives (TP), false positives (FP) and the resulting precision using micro-averaging. A bit more than one third of all sentences fully expressed the desired relationship. In order to assess the ranking quality of the system, we computed the mean average precision (MAP) and compared it with three alternative ranking scenarios:

**Table 7.** Evaluation results of task 2 including mean average precision (MAP)

Criterion	TP	FP	Precision	MAP	Worst	Random	Best
Full	316	490	39.2%	49.0%	31.7%	46.5%	74.2%
Relaxed	429	377	53.2%	62.1%	45.9%	58.4%	80.4%	
Context	496	310	61.5%	68.9%	55.2%	65.7%	83.5%

- **Worst**: All true positives are ranked after all false positives.
- **Random**: We randomly reordered the results 2000 times and computed the average MAP for all these variants.
- **Best**: All true positives are ranked before all false positives.

Table 7 shows that the system performs consistently better than random ranking. In maximum, 3.7 percentage points improvement could be reached for the relaxed criterion compared to random ranking. The best ranking is 25% higher for the strictest criterion (fully supportive) and 18% and 15% for the relaxed criterion and the context criterion respectively. These results show that there is some capacity for improvement. The resulting annotated corpus is published as BEL_Sentence_Classification corpus (see (11) for further details), since it provides positive as well as negative evidences for the given BEL statements.

## Participating systems

In this section, we describe important aspects of the contributing systems. For task 1, we had five participating systems. The best systems integrated and adapted existing state-of-the-art components for biomedical text mining and turned their output into the requested BEL format. Two of the participating teams (referred to as s1 and s2 in the previous section) decided not to submit a system description, and were therefore omitted from this survey.

System s3 (24) decomposes the problem of task 1 into three separate modules: (a) a natural language processing step which includes syntactic parsing and rule-based coreference resolution, (b) a state-of-the-art event extraction system (TEES) which produces GENIA event structures as known from the BioNLP 2009 shared task, (c) an existing BEL generation module which translates the GENIA event structures into BEL statements. Their system relies on the BANNER named entity recognition system, which is limited to proteins and genes. This explains the performance gain of the system when gold entities were provided to the participants. The coreference module could not improve results on the test data, although a small improvement could be seen on the training data. However, given that in task 1 the input for BEL statements consisted of single sentences this should not be taken as a general argument against coreference resolution in BEL statement generation.

System s4 (25) uses four processing steps: (a) named entity recognition for DNA, RNA, proteins, cell lines and cell types is performed by a CRF-based component; another NER system is used for chemical abundances, and another dictionary-based component recognizes GO terms and diseases. In step (b), the identified named entities are normalized into their database identifiers using the Entrez homolog dictionary. In step (c), functions are classified by keywords appearing in the context of entities. In step (d), causal relationships are classified via the output of a biomedical semantic role labeler.

The approach followed by s5 (26) is centered upon a rule-based semantic parser capable of handling complex syntactic structures involving connectives, events and anaphoras. It uses a frame-based approach, with 15 verb categories and >70 verbs. The structures produced by the semantic parser are then translated into BEL annotations, by mapping specific biological events (e.g. phosphorylation) to BEL functions, and the core causal relations (increase, decrease) to BEL relations. In several cases structures generated by the parser have to be dropped as they do not have an equivalent in BEL syntax.

Entity extraction is based on an ensemble of NER systems (PubTator and beCAS, plus an in-house developed dictionary lookup system). The different systems perform differently on some entity classes (for example the authors report that they give preference to PubTator for genes/proteins, chemicals and diseases, while preferring beCAS for GO terms). When the confidence in an annotated entity or namespace is low, it is replaced by the placeholder PH:Placeholder. Such approach however causes a low performance in stage 1 (overall *F*-score 18.2%). When using the gold standard entities provided by the organizers (stage 2), the performance of the system improves significantly (overall *F*-score 25.6%).

The results on extracting functions are relatively poor (around 30% in the primary evaluation, around 50% in the secondary evaluation) and are considered as the main cause of the overall low performance. The strength of the system lies in relation extraction (72.7% *F*-score in stage 1, 83.4% in stage 2) with a very high precision (up to 94.4% in stage 2) with a reasonable recall (74.8% in stage 2). There is a performance gain of 13% going from stage 1 to stage 2, when gold standard entities are provided. The main causes of errors can be tracked down to named entity recognition and function identification. Additionally, the system lacks the ability to extract long distance relationships and recursive relations, plus certain semantic inferences.

The system participating on task 2 performs two main steps: a retrieval and a reranking step. For each BEL statement, the retrieval components gathers relevant documents from PubMed and PubMed Central. The ranking component identifies the significant evidence texts and ranks their relevance. Further details and evaluation results have been described by Rastegar-Mojarad et al. (27).

## Conclusions

The BEL track at BioCreative 2015 offered a novel platform for the evaluation of text mining systems capable of dealing with BEL statements. BEL provides a compact yet perspicuous format of knowledge representation in the biomedical fields, which combines information at several levels: from named entities, to functions, to relationships. BEL provides all these different levels of information from the original evidence text in a compact and human-understandable representation. However, text mining systems need to unpack this complexity, in order to be able to automatically construct BEL statements from text. We have designed an evaluation framework which takes this complexity into account, and attempts to give credit to systems capable of finding BEL fragments which could be combined into the full statement.

The participants in task 1 have shown that text mining systems can reach satisfactory levels of performance in the extraction of BEL fragments from text. Although significant scope for improvement still remains, some of the systems could already be used to provide valuable input for a semi-automated curation environment. Additionally, we have shown that a hypothetical ensemble system, which accepts a BEL statement if at least two different systems predict it, leads to even more valuable results.

As for task 2, although only one group participated, the problem of finding supporting evidence for biological statements in a large body of biomedical texts remains crucial. Additionally, the task provides the text mining community with large-scale training material which can be used for future development and evaluation.

*Conflict of interest*. None declared.
